# Multi-species consumer jams and the fall of guarded corals to crown-of-thorns seastar outbreaks

**DOI:** 10.12688/f1000research.13118.2

**Published:** 2018-03-02

**Authors:** Mohsen Kayal, Jane Ballard, Mehdi Adjeroud

**Affiliations:** 1 UPVD-CNRS, Centre de Formation et de Recherche sur les Environnements Méditerranéens (CEFREM), UMR 5110, Perpignan, 66860, France; 2 Centre de Recherche sur les Ecosystèmes Marins (CREM), Port-Barcarès, 66420, France; 3 National Estuarine Research Reserve Association (NERRA), Wells, ME, 04090, USA; 4 Institut de Recherche pour le Développement (IRD), UMR-9220 ENTROPIE, Perpignan, 66860, France

**Keywords:** Predator outbreak, Acanthaster, Mutualistic defense, Guardian crab, Trapezia, Mixed-species predator guild, Trophic cascade, Density dependence.

## Abstract

Outbreaks of predatory crown-of-thorns seastars (COTS) can devastate coral reef ecosystems, yet some corals possess mutualistic guardian crabs that defend against COTS attacks. However, guarded corals do not always survive COTS outbreaks, with the ecological mechanisms sealing the fate of these corals during COTS infestations remaining unknown. In August 2008 in Moorea (17.539° S, 149.830° W), French Polynesia, an unusually dense multi-species aggregation of predators was observed feeding upon guarded corals following widespread coral decline due to COTS predation. Concurrent assaults from these amplified, mixed-species predator guilds likely overwhelm mutualistic crab defense, ultimately leading to the fall of guarded corals. Our observations indicate that guarded corals can sustain devastating COTS attacks for an extended duration, but eventually concede to intensifying assaults from diverse predators that aggregate in high numbers as alternative prey decays. The fall of guarded corals is therefore suggested to be ultimately driven by an indirect trophic cascade that leads to amplified attacks from diverse starving predators following prey decline, rather than COTS assaults alone.

## Introduction

**Figure 1.**
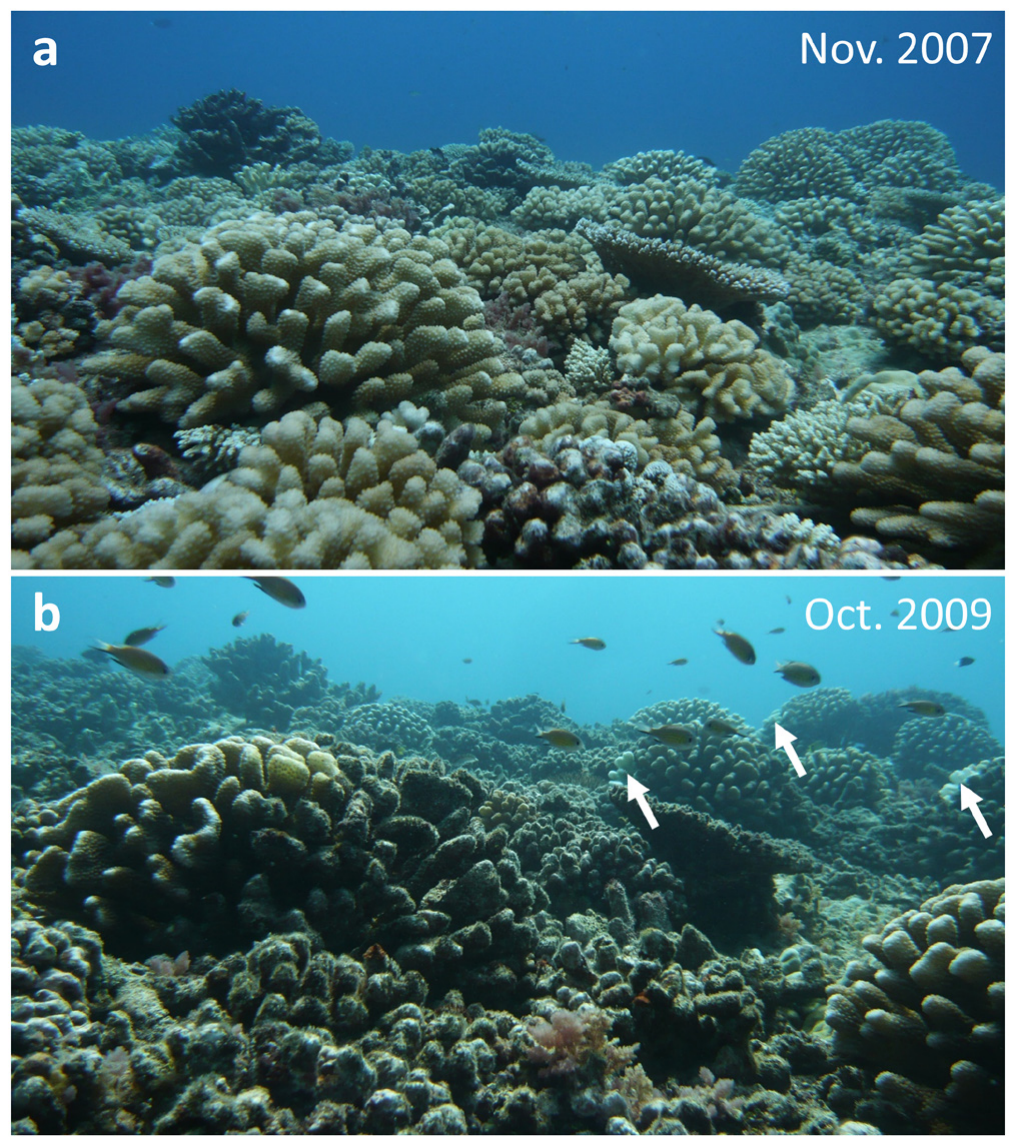
Widespread coral decline and survival of guarded corals that partially or fully resisted seastar predation. Pictures were taken at 6 m depth on Tiahura reef in Moorea, French Polynesia, before ( **a**) and after ( **b**) this location was invaded by crown-of-thorns seastar (COTS) swarms. Surviving guarded corals typically measured above 60 cm in diameter. Arrows in **b** indicate white feeding scars characteristic of recent COTS predation on several of the guarded coral colonies ( *Pocillopora eydouxi*).

Identifying ecological processes that drive species trajectories is a prerequisite for ecosystem management. However, community dynamics are sometimes governed by unexpected, indirect interactions and complex emergent properties that can cause runaway responses and abrupt ecological shifts ( Silliman *et al.*, 2013; Terborgh & Estes, 2010). Outbreaks of the coral predator crown-of-thorns seastar (COTS) cause widespread coral mortality across the Indo-Pacific Ocean ( Pratchett *et al.*, 2014) with often drastic impacts on diverse reef communities ( Kayal *et al.*, 2012). However, some coral species possess mutualistic allies that can deter COTS predation. In particular, trapeziid crabs and alpheid shrimps inhabiting large pocilloporids are known for their ability to effectively defend their host corals from COTS assaults ( Glynn, 2013; McKeon & Moore, 2014), although guarded pocilloporids do not always survive COTS outbreaks ( Leray *et al.*, 2012; see Figure 1). Despite increasing understanding of factors determining coral susceptibility to COTS predation ( Glynn, 1976; Kayal *et al.*, 2011; Kayal & Kayal, 2017; Pratchett, 2001; Rouzé *et al.*, 2014), the processes sealing the fate of guarded corals during outbreaks have remained unknown. Here we provide insights into the ecological mechanisms underlying the fall of guarded corals during predatory COTS outbreaks.

## Methods

Our observations were performed at the peak of an intense crown-of-thorns seastar (COTS) outbreak that decimated coral communities around the island of Moorea (17.539° S, 149.830° W), French Polynesia, between 2003 and 2010. General patterns in propagation of COTS swarms around the island, and impacts on corals and other reef communities were described by Kayal *et al*. (2012); Kayal *et al*. (2017). Here, we provide complementary observations that unveil processes leading to the fall of large (typically above 60 cm diameter) pocilloporid assemblages that benefit from “anti-COTS” mutualistic defense, the so-called guarded corals. In Moorea, these assemblages are dominated by *Pocillopora eydouxi*, a species that hosts trapeziid crabs and alpheid shrimps able to deter COTS predation ( Glynn, 2013; Leray *et al.*, 2012; McKeon & Moore, 2014; Figure 1). Our observations were performed using SCUBA on the outer reef slope at Tiahura where the COTS outbreaks in Moorea were initiated and had particularly detrimental impacts ( Kayal *et al.*, 2012).

## Results and discussion

**Figure 2.**
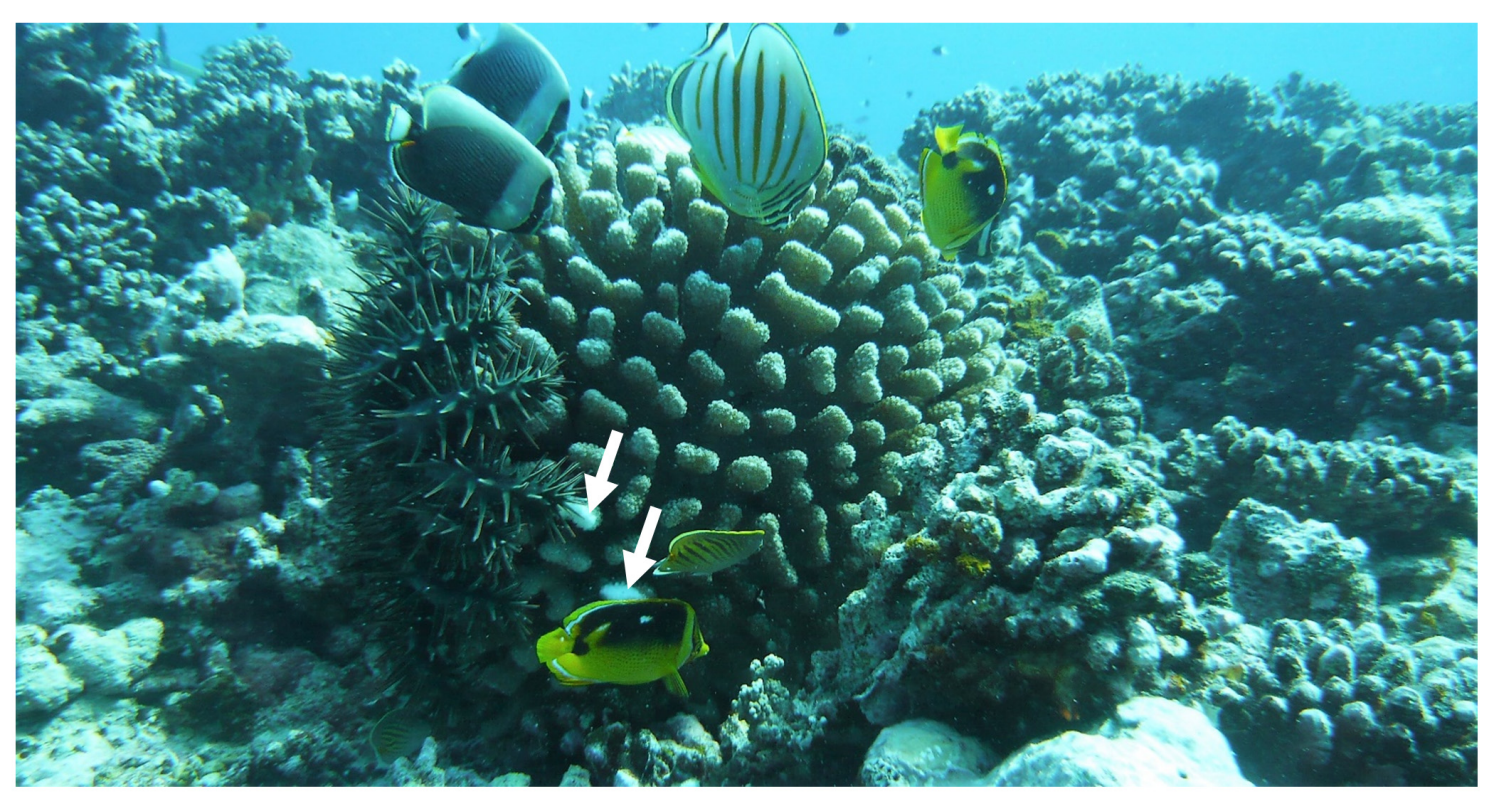
Aggregation of a diversified guild of 10 macro-predators simultaneously feeding upon a guarded coral. This aggregation was observed following widespread coral decline (note the absence of live coral in the background) in August 2008 at 12 m depth on Tiahura reef in Moorea, French Polynesia. The guarded coral measured approximately 70 cm in diameter. The predator guild was composed of a crown-of-thorns seastar (COTS) and nine butterflyfishes from species *Chaetodon ornatissimus*, *C. pelewensis*, *C. quadrimaculatus*, *C. reticulatus*. Arrows indicate white feeding scars characteristic of recent COTS predation on the guarded coral ( *Pocillopora eydouxi*).

In August 2008 at 12 m depth on Tiahura reef, we observed an unusually dense aggregation of coral-eating butterflyfishes jamming around guarded pocilloporids, the last coral bastions that had yet resisted swarms of the predatory seastar ( Figure 2, Supplementary Image 1). Widespread coral decline had previously wiped out much of resident populations of coral-feeding butterflyfishes ( Kayal *et al.*, 2012), pushing starving survivors to aggregate around the guarded corals. While butterflyfishes were increasingly observed to gather around guarded corals as the COTS outbreak progressed around the island, the aggregation of 9 butterflyfishes within a single square-meter (9 fish.m ^-2^), as captured in Figure 2, was particularly surprising. Density of the coral-feeding butterflyfish assemblage on this reef location had dropped to the much lower average value of 4.3±0.9 SE fish.200m ^-2^ following the COTS outbreak (surveyed in June 2008, equivalent to 0.02 fish.m ^-2^). The observed aggregation thus represented a more than 400-times concentration of the predation pressure exerted by the butterflyfishes, and was targeting a guarded pocilloporid that was already under attack by COTS ( Figure 2, Supplementary Image 1).

Guarded pocilloporids in Moorea have shown the ability to resist devastating COTS predation for several years ( McKeon & Moore, 2014; Figure 1). However, concurrent assaults from such locally amplified, mixed-species predatory guilds likely overwhelm the ability of trapeziid crabs and other exo-symbionts to defend host pocilloporids, ultimately causing the fall of guarded corals. Indeed, coral occupation by mutualist communities is determined by strict rules of territoriality and competition ( Glynn, 2013; Leray *et al.*, 2012), which limits the abundance of inhabiting guardians in host colonies, and therefore their ability to sustain predatory assaults. The relative contribution of the butterflyfishes, as compared to COTS, to the death of guarded corals at this stage remains unclear. Further research is needed to quantitatively evaluate the aptitude of coral mutualists to withstand attacks from mono- versus multi-specific predators at different abundances. This particularly applies when the predator guilds involve specialized coral-feeding species from distant phyla with different feeding modes, such as fishes that sample polyps through repeated rapid bites and seastars that consume large portions of coral tissue over extended amount of time. Coral decline has already been identified as an engine of COTS movements and prey selection during outbreaks ( Kayal *et al.*, 2011; Kayal *et al.*, 2012; Silliman *et al.*, 2013). Our observations suggest that further cascading effects include aggregating diverse predators in numbers surpassing mutualistic defenses, eventually leading to the collapse of guarded corals. We therefore advocate the importance of controlling COTS outbreaks at the earliest stages, before trophic cascades could lead to a runaway collapse of coral communities.

## Data availability

The data referenced by this article are under copyright with the following copyright statement: Copyright: © 2018 Kayal M et al.

Data associated with the article are available under the terms of the Creative Commons Zero "No rights reserved" data waiver (CC0 1.0 Public domain dedication). 

*All data underlying the results are available as part of the article and no additional source data are required*.

## Supplementary material

**Supplementary Image 1.** Additional photographs capturing the predatory guild aggregating around a guarded coral following widespread coral decline. Pictures ( **a**, **b**) were taken in August 2008 at 12 m depth on Tiahura reef in Moorea, French Polynesia, at the peak of an intense outbreak of the coral-eating crown-of-thorns seastar (COTS). The observed macro-predator aggregation was composed of one COTS and nine individuals from resident butterflyfish species *Chaetodon ornatissimus*, *C. pelewensis*, *C. quadrimaculatus*, *C. reticulatus* (see Figure 2). White feeding scars characteristic of recent COTS predation can be seen on the guarded coral ( *Pocillopora eydouxi*).
