# Supplementary figures and images for: Multi-species consumer jams and the fall of guarded corals to crown-of-thorns seastar outbreaks
Source: F1000Res. 2018 Mar 2;6:1991. Originally published 2017 Nov 13. [Version 2] doi: 10.12688/f1000research.13118.2 (PMC5806057; doi:10.12688/f1000research.13118.2)

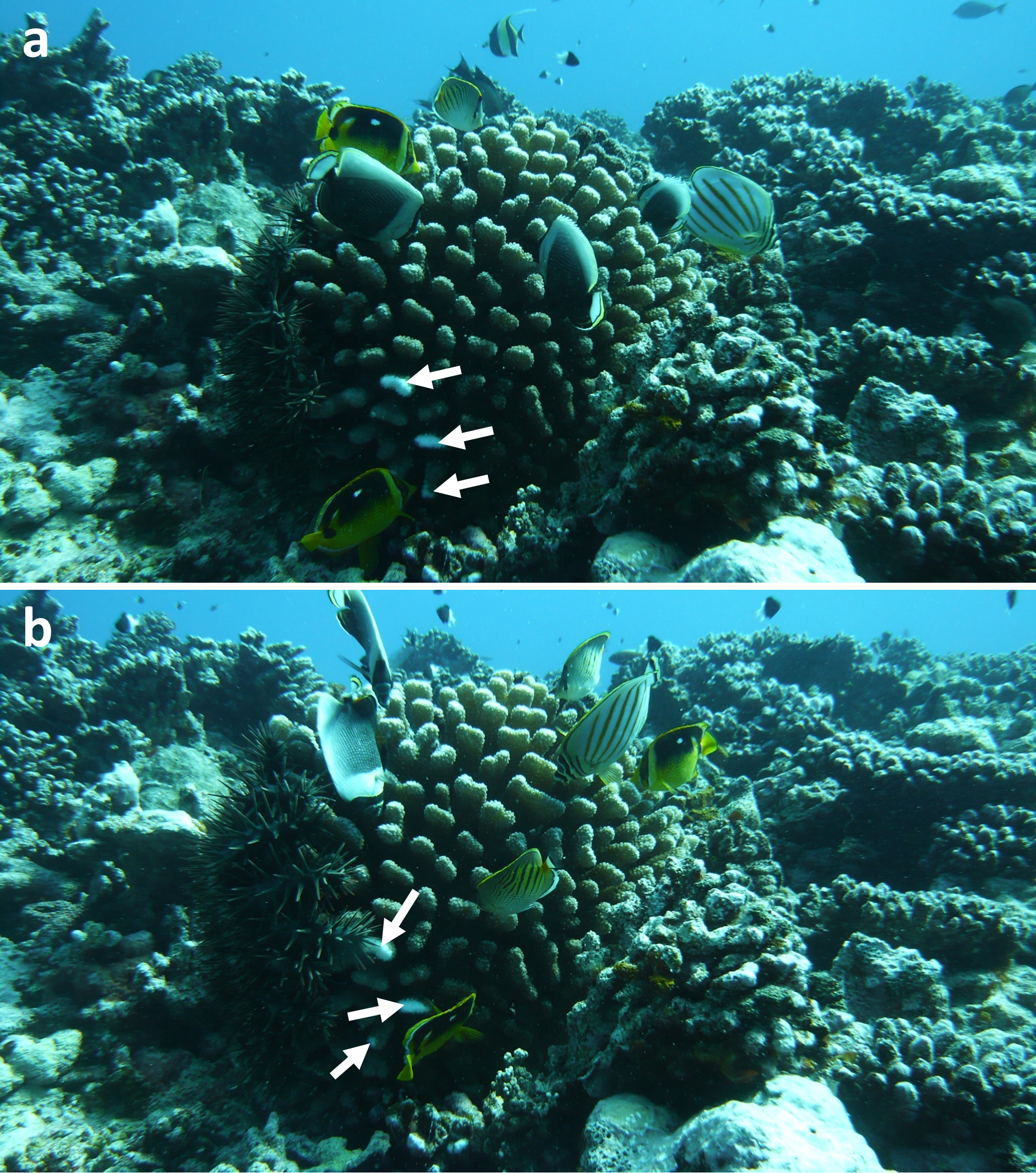

Supplement: Supplementary file 1 [file f1000research-6-15370-s0000.tgz › a55f2cd1-20e2-4e6a-8d34-39b6ea0a44ba.tif]
