# Supplementary material for: Multi-species consumer jams and the fall of guarded corals to crown-of-thorns seastar outbreaks
Source: F1000Res. 2018 Mar 2;6:1991. Originally published 2017 Nov 13. [Version 2] doi: 10.12688/f1000research.13118.2 (PMC5806057; doi:10.12688/f1000research.13118.2)
Supplement: Peer review history

# Peer review: Referee response for version 1

**DOI:** 10.5256/f1000research.14230.r30512

**Published:** 2018-02-07

**Authors:** Ciemon Frank Caballes

**Affiliations:** 1 ARC Centre of Excellence for Coral Reef Studies, James Cook University, Townsville, Qld, Australia

This is an interesting observation of a relatively high concentration of a multi-species predator guild on corals that were initially spared from crown-of-thorns seastar (COTS) predation. The ultimate demise of "guarded" Pocilloporids may have been due to a high density of starving COTS (at the peak of an outbreak) feeding on whatever coral was left and overwhelming mutualistic crabs in the process. The overall impact of the butterflyfish, in terms of coral mortality, was most likely lower compared to COTS.

 It is unclear whether this was a widespread occurrence or a one-time observation. A brief description of the feeding behaviour of COTS and butterflyfishes (relative contribution to coral mortality), as well as the defensive behaviour of Trapeziid crabs will be useful.

 METHODS: Change *Pocilloporida eydouxi *to *Pocillopora eydouxi.*

 SUPPLEMENTARY IMAGE 1: Feeding scars are not clear in the pictures.

I have read this submission. I believe that I have an appropriate level of expertise to confirm that it is of an acceptable scientific standard.

# Peer review: Referee response for version 1

**DOI:** 10.5256/f1000research.14230.r27894

**Published:** 2017-12-12

**Authors:** Peter W. Glynn

**Affiliations:** 1 Rosenstiel School of Marine and Atmospheric Science, Department of Marine Biology and Ecology, University of Miami, Miami, FL, USA

The alpheid shrimp guard, *Alpheus lottini*, also should be noted as defending pocilloporid corals from COTS attacks.  This shrimp guard occurs world-wide on pocilloporid corals. 

 It would also be worth noting the defensive behaviour, if any, of the crustacean guards toward the fish corallivores.

 ‘White feeding scars’ are referred to in Fig. 1 and Fig. 2 (supplementary image).  These are difficult to make out in the photographs.  I suggest adding arrows to make these easier to see.  Also, it would be useful to know the approximate diameters of the *P. eydouxi* colonies.

I have read this submission. I believe that I have an appropriate level of expertise to confirm that it is of an acceptable scientific standard.
